# Medical Association of Central New York

**Published:** 1880-01

**Authors:** Charles E. Rider

**Affiliations:** Secretary


### MEDICAL ASSOCIATION OF CENTRAL NEW YORK.

Twelfth semi-annual meeting of the Medical Association of Central New York, held in Association Hall, Syracuse, Nov. 18, 1879.

The President, Prof. Frederick Hyde, M. D., of Cortland, called the meeting to order, and made a few appropriate remarks, congratulating the Society on its past success and continued prosperity.

The minutes of the last annual meeting were read by the Secretary, Dr. C. E. Rider, of Rochester, and adopted.

The President announced the following committees:

On Credentials—Drs. Alfred Mercer and M. B. Fairchild, of Syracuse, and J. O. Roe, of Rochester.

On Business—Drs. I. N. Goff, of Cazenovia, A. G. Crittenden, of Clifton Springs, and T. Dimon, of Auburn.

Dr. W. W. Potter, of Batavia, read a paper on "Rectal Alimentation," giving in full a report of a case where such means were used, and laying down explicit indications for rectal alimentation in cases of nausea from gravid uterus.

Dr. Creveling, of Auburn, doubted whether nutritious substances so injected, passed above the colon, or were digested.

Dr. Crittenden mentioned a case where mercurial inunction seemed to relieve a severe nausea dependent upon pregnancy.

Dr. J. O. Roe read a paper on pharyngeal tuberculosis, giving his minutes of a case which ended in death from pulmonary disease.

Dr. Creveling mentioned a case in his practice where the edges of the pharyngeal ulcer projected into the pharynx, contrary to the rule, and where the larynx has subsequently became involved.

Dr. Crittenden read a paper on colloid degeneration of the omentum, detailing a case which had occurred in his practice. (See Clinical Reports.)

Dr. M. D. Benedict, of Syracuse, mentioned a case of omental cancer which he had observed in the army.

Dr. C. C. Bates, of Auburn, had also seen such a case in a female, where an early diagnosis was difficult on account of the sex.

Adjourned for dinner.

AFTERNOON SESSION.

Dr. D. C. Crumb, of Chenango County, stated that he had observed three cases of cancerous disease of the liver in one family.

The question of hemorrhage from paracentesis abdominis arose, and Drs. Mercer, of Syracuse, and Gilmore, of Cayuga County, cited cases.

Dr. Starr, of Rochester, quoted Dr. Syme, of Edinburgh, who mentions some six cases where dangerous hemorrhage followed paracentesis.

Dr. Darwin Colvin, of Clyde, then read a paper on prolapse of the ovary, citing several cases where such a diagnosis was made, and outlining the methods of treatment.

Dr. E. Van DeWarker, of Syracuse, did not think the displacement mentioned was a rare one. The extreme mobility of the ovaries predisposes to such a result. He cited a paper by Munde as a valuable one.

Prof. W. W. Porter, of Syracuse, recalled six cases of this disease in his practice, five of which were on the left side.

Dr. J. E. Carr, of Jordan, then exhibited, as a pathological specimen, an exudate from the uterus.

Dr. A. S. Coe, of Oswego, read a paper on hydatid moles. (See Clinical Reports.)

Dr. C. S. Starr, of Rochester, read a paper on Bronchocele.

Dr. J. E. Carr said that he treated goitre with as much confidence as ague. The remedies are iodide of potassium internally, and tinct. iodine externally. He never saw a case that did not yield, if so treated, during its early stages.

Dr. Craig, of Churchville, agreed substantially with Dr. Carr.

Dr. Didama, of Syracuse, remarked that some cases do well with iodine, while many cases do not yield to any treatment.

Dr. Hoxie cited the case of a man with goitre treated with iodine. The tumor increased, although treatment was continued for a year.

Dr. Colvin said he had treated goitre for 35 years, and never saw a case get entirely well. The tumor will perhaps diminish for awhile, but is prone to increase again. He did not believe that any remedy could be considered a specific. Some cases improve without treatment.

Dr. Starr said he had spent some time in Vienna, and on the slopes of the Alps where cases of goitre abounded. European physicians state that cases of over two years' duration are seldom cured.

Dr. R. W. Pease, of Syracuse, did not doubt that good might come from treatment. He thought well of electrolysis in some cases, but even if temporarily relieved, the disease was apt to return.

Dr. Hyde thought that the general health should be especially attended to in such cases, especially where the subjects were young women.

Dr. Goff saw a case cured by the hypodermic injection of ergot.

Dr. Van DeWarker, of Syracuse, then read a paper on the operation for laceration of the cervix uteri, and exhibited his instrument by which many difficulties of the operation are overcome.

Dr. Clark, of Oswego, then read an elaborate paper on puerperal convulsions. The treatment recommended was the hypodermic injection of morphia. He injected fearlessly a grain and a half for the first dose. Given in divided doses, morphine was impotent—its action should be intense.

This proposition elicited much discussion, some gentlemen supporting warmly the views of Dr. Clark.

Adjourned to meet in Rochester, May 18, 1880.

Charles E. Rider,
Secretary.

